# Mitigating the Effects of Maternal Loss on Harbour Seal Pups in Captive Care

**DOI:** 10.3390/ani14223264

**Published:** 2024-11-13

**Authors:** Susan C. Wilson, Rhiannon Alger

**Affiliations:** Tara Seal Research, Downpatrick BT30 9QN, UK; rhiannonalger2@gmail.com

**Keywords:** harbour seal, orphan pups, captive care, sensitive period, social interaction

## Abstract

Stranded newborn “orphan” harbour seal pups entering captive care are often maintained for some weeks in isolation, mainly as a precaution against the spread of infection. However, this practice raises concerns for the welfare and normal socialisation of pups, who normally spend their first post-natal weeks close to their mothers and other seals. The present study recorded and described the behaviour of six paired orphan pups in rehabilitation up to about five weeks of age, provided with free access to water and haul-out areas. The occurrences of resting, following, nosing and body contacts, and aquatic play were recorded and compared qualitatively and quantitatively with the same behaviours of free-living pups with their mothers. The pups entered the water every day, although more often from about 2.5 weeks of age. They displayed to each other the same behaviours that free-living pups display to their mothers, although they engaged in relatively more physical contact, body nosing, and aquatic play. The study has shown that orphan pups maintained in pairs with free water access can act reciprocally as mother substitutes, thereby promoting species-typical primary socialisation and welfare during their early days of captive care.

## Simple Summary

Harbour seal pups in the wild are highly social, initially just with their mothers and then, after about three weeks, with other pups. They rest close together onshore, while their active time is spent in the water, following and interacting. This social contact in the first few weeks after birth is thought to be essential for social development, because after 4–6 weeks of age, the pups gradually disperse to sea while they learn to fish. However, stranded “orphan” neonate pups taken to rehabilitation centres are often kept singly, often in dry pens, for some weeks, raising concerns about their welfare and social development. In this study, orphan pups were kept in pairs with free water access and their behaviour recorded and compared with that of wild pups with their mothers up to five weeks of age. The study was able to demonstrate that the paired pups self-regulated their time in the water and acted as mother substitutes to each other, displaying qualitatively the same behaviours as wild pups would towards their mothers. Pairing orphan pups can thus mitigate maternal loss to some extent and help towards normal social development.

## 1. Introduction

**Figure A1:**
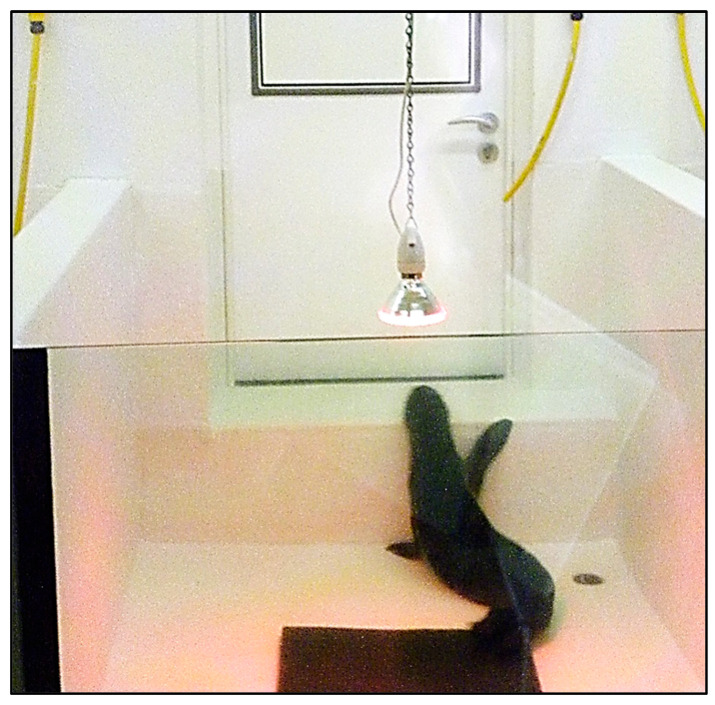
Orphan harbour seal pup isolated in dry pen with heat lamp overhead (seal rehab centre in UK).

Neonatal harbour seal (*Phoca vitulina*) pups are sometimes found stranded alone and believed to be permanently separated from their mothers [1]. The rescue, captive care, and subsequent release of such “orphan” pups have become frequent practices in North America and western Europe [1,2,3]. The newly rescued pups are often isolated in ‘quarantine’ pens (Figure A1) for some weeks to avoid the cross-infection of diseases [3,4,5,6].

This typical quarantine procedure raises the concern of the young pups being deprived of the social contact, physical activity, and mental stimulation that free-living neonates would naturally experience. A harbour seal neonate in the wild is highly precocial and may spend up to three-quarters of its time in the water with its mother [7,8,9], with the proportion of time increasing from ~35% by neonates to ~75% at 20 days [10,11]. During the 3–4-week nursing period, the pup is continually in contact and interacting with its mother in the water or resting onshore close by other mother–pup pairs, and the strong bond with its mother is considered to be essential for survival [12,13,14,15,16]. After weaning, pups interact socially with other pups up to 5–7 weeks of age before dispersing to foraging areas [17,18,19,20]. It is therefore likely that the post-natal period prior to this dispersal approximates a sensitive period for species-typical primary socialisation [21,22,23].

Maternal separation and isolation in infant mammals have long been recognised to have a short- and long-term effect on the infant’s brain and physiological development [24,25]. The social deprivation or isolation of infants of domestic and laboratory mammals (rodents, monkeys, domestic lambs, dairy calves) during this sensitive period has been shown to result in neurological abnormalities in the pre-frontal cortex [26,27,28,29], resulting in later social behaviour deficits and reduced levels of maternal care [30]. Reduced maternal care by rehabilitated seals in adulthood might therefore result in poor survival of their pups.

Impaired later cognitive function of isolated infants has also been demonstrated in domestic and laboratory animals [24,31]. Tests carried out on maternally separated domestic calves have shown that individually housed calves show poor performance on later reversal learning tests compared with calves housed in pairs [32,33]. Experiments on spatial reversal learning with adult captive-bred harbour seals have demonstrated their excellent performance [34], considered to be highly advantageous to optimal foraging decision making with changing conditions in the wild [35,36]. If social isolation during the pups’ first 3–4 post-natal weeks should have a detrimental effect on their later flexible spatial learning ability, this could affect their foraging abilities post-release.

There has long been concern that rehabilitated pups may behave differently from their wild peers and survive less well after release. Seals need well-developed cognitive abilities related to spatial reversal learning, important for effective foraging strategies [34]. It is likely that pups after release will need to learn to follow hydrodynamic trails in the wake of fish prey [37], and learn to orientate using geometrical relationships between landmarks [38], and even to navigate by the stars [39]. However, tracking studies of orphan harbour seal pups after release—compared with weaned wild pups (in the inland waters of Washington and British Columbia)—have indicated that they may travel and disperse nearly three times as far as wild pups [40,41], suggesting that they may be disorientated due to environmental naivety, undeveloped navigation skills, or possibly a cognitive deficit.

Previous studies of orphan harbour seal pups in rehabilitation (rehab) have focused on greater pup weight gain and relatively short time in rehab as factors favouring post-release survival [42,43,44], while a study of blood oxygen storage capacity found that it increased with time the pups spent submerged [45]. However, orphan seal pups’ early social development in rehab has yet to be considered in any detail [16].

Maternal loss and isolation may also result in a sustained depressive state, as demonstrated in infant guinea pigs (*Cavia porcellus*) [46]. However, giving maternally separated infant rats a companion animal has been found to reverse elevated glucocorticoid levels resulting from the separation [47]. For maternally separated dairy calves, contact with another calf results in behavioural indicators of a positive affective state such as play [48,49]. A study of glucocorticoid levels in orphan harbour seal pups suggested that levels declined with time for pups with water access and a companion pup, although the study concluded that behaviour studies rather than stress hormone measurement are needed to assess pup well-being [50].

Previous informal behavioural observations at Tara Seal Research (TSR) of six pairs of neonate orphan harbour seal pups maintained with water access have indicated that the pup pairs appeared to form a strong social bond, resting close together and playing together, suggestive of a positive affective state [42]; https://www.sealresearch.org/rehab-pups/rehab-diary, accessed on 22 September 2024. This already suggested that pairing orphan harbour seals during rehab may work effectively to mitigate maternal separation by social buffering.

For the present study, we video-recorded the behaviour of three further cohabiting orphan pup pairs with the primary objective of making a direct comparison between the behaviours of these rehab pup pairs and the behaviours of wild pup–mother pairs. The aim of the video analysis was to enable the direct comparison by following, as closely as possible, the methods used to quantify wild harbour seal pup–mother behaviour in a previous study [16] (summarised in Appendix A), and thereby document the potential success of maternal substitution with a companion pup.

## 2. Animals and Methods

### 2.1. Pup Stranding, Rescue, Captive Care

**Figure 1:**
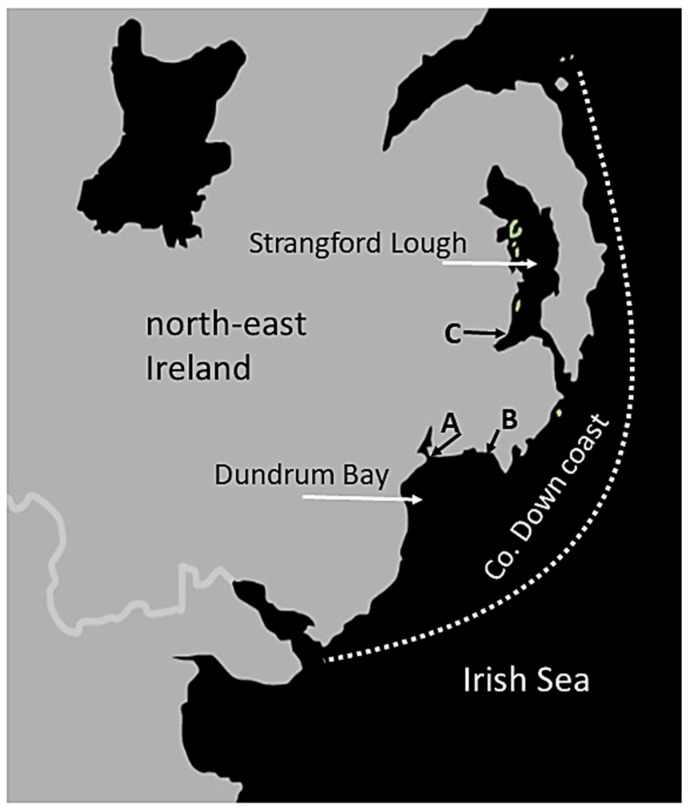
The Co. Down coast of north-east Ireland, showing the locations of stranding of pups (**A**) M&M (“Maxi” and “Mini”), 54.235 N, 5.8166 W; (**B**) C&P (“Coral” and “Pearl”) and E (“Earendil”), 54.2328 N, 5.7595 W, all in Dundrum Bay; and (**C**) U (“Ula”; 54.401 N, 5.6398 W) in Strangford Lough (Table 1).

The six harbour seal pups in this study had all been stranded during the peak of the summer pupping season on the Co. Down coast of north-east Ireland (Figure 1).

The pups were taken into captive care for rehab at TSR (located close to the stranding position of pup “Ula” (U)) by the author (SW). All six pups had been identified, according to a protocol [1], as being no more than a few days old *and* as having been permanently separated from their mother (Table 1). Two of the pups (“Maxi” and “Mini”) were found together at stranding in 2013, while the other four pups were stranded separately, “Ula” and “Earendil” in 2014 and “Coral” and “Pearl” in 2016. These six pups were kept in pairs, with each pup introduced to its partner as soon as the second pup was rescued (Table 1).

The rehab enclosure consisted of an outdoor paved yard measuring approximately 6.1 × 1.4 m. The average temperature in Co. Down during July and August is 18 °C, with a minimum of 12°; https://weather-and-climate.com/newcastle-down-county-gb-August-averages, accessed on 9 October 2024. The yard contained two mini trampolines with approximately a 93 cm diameter, one at each end of the yard. The pup pair Maxi and Mini (M&M) had a paddling pool (approximately 1 m diameter) beside each trampoline. The subsequent pup pairs, Ula and Earendil (U&E) and Coral and Pearl (C&P), had a paddling pool at one end of the yard and a bath (approximately 170 cm long and 35 cm deep) at the other end, with the trampoline adjacent to enable the pups’ independent access to the bath. Pools and bath were filled with tap water at ~14 °C.

The pups were fed five times a day with a minimum 4 h interval for the first two weeks and four times a day thereafter. Since orphan harbour seal pups in rehab will rarely suckle from a bottle, feeding throughout the rehab period was by a liquid formula through a silicone tube inserted orally into the stomach. Feeding was as described for the previous 15 orphan pups cared for by the present author (SCW) [42]. The formula was made up from Zoologic Milk Matrix 30/55 milk powder (Pet-Ag, Hampshire, IL, USA), containing a minimum of 55% fat and 30% protein. Each milk feed was supplemented with homogenised canned fish, a small amount of mixed fish oil, and digestive enzymes (Essential Enzymes, Source Naturals, U.S.A. (www.sourcenaturals.com)). Each feed was 250–300 mL for the first two weeks, and up to 400 mL thereafter. The pups were weighed just before feeding, usually once every two days, by holding the pup while standing on digital bathroom scales. Cleaning of the yard and replacing of the water was usually carried out immediately after feeding. Otherwise, the pups were left undisturbed as much as possible between feeds. The total time in rehab before their release was approximately seven weeks for each of the six pups.

### 2.2. Rehab Pup Behaviour Recording

**Figure 2:**
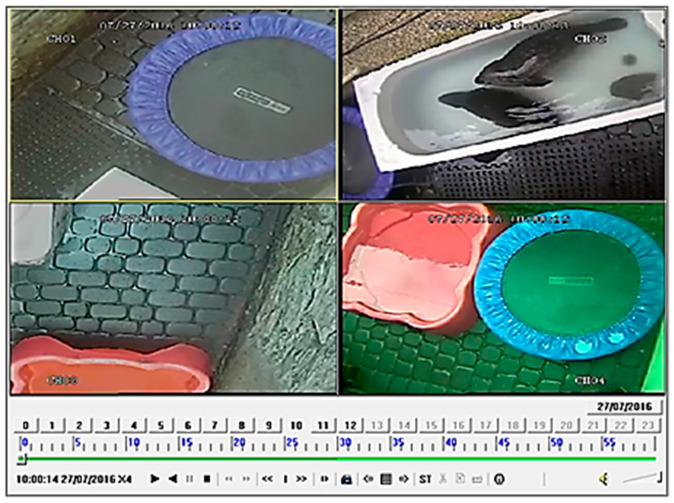
Example of CCTV recording windows and play-back system.

The behaviour of the three pairs of rehab pups was recorded by continuous CCTV (“Storage Options DIY Home CCTV Kit”), using two overhead IR cameras (“Storage Options” CCTV package) in 2013, and a further two cameras (“Swann Pro 770”) in 2014 and 2016. The cameras were attached to the wall at either end of the yard and attached to a wire strung between the two walls at approximately a 2 m height. This system was able to record in compressed format continuously 24 h/day for 50 days without overwriting. Recordings were saved daily onto a memory stick, transferred to a hard drive, and viewed using “Playback”v.2.3.0.4 software (Figure 2). An attempt was made to use a newer DVR system (“SentientPro, Sen108”) for C&P in 2016, but this was discontinued due to technical problems (resulting in overwriting of the first week’s record); the remainder of the 2016 recording reverted to the “Storage Options” DVR.

The rehab pups’ location in the yard was classified as in the *water* (in either the bath or the paddling pool), *water’s edge* (any part of the body partially in the water or pups interacting along the edge of the pool when one is in the water while the other is outside of the pool), and *dry zone* (both pups outside of the bath or paddling pool, on the trampoline, or on yard paving).

The immersion of seal pups in water should result in the pup regulating its core body temperature by vasoconstriction at the body surface, resulting in a significant drop in surface body temperature and recovery when the pup is again dry [51]. Because the pups at TSR were allowed free access to water, their thermoregulation capacity was assessed by recording the surface body temperatures of both C&P in 2016 (using a hand-held *Flir i3* camera, Teledyne FLIR, Wilsonville, OR, USA) opportunistically when they were newly emerging from the water and when they were dry. Each *Flir* photograph displays the surface body temperature of the body area photographed. The body areas photographed included the nostrils, side of neck, flank, back, hind flippers, and fore flippers.

### 2.3. Behaviours Recorded, Data Sampling and Processing

**Figure 3:**
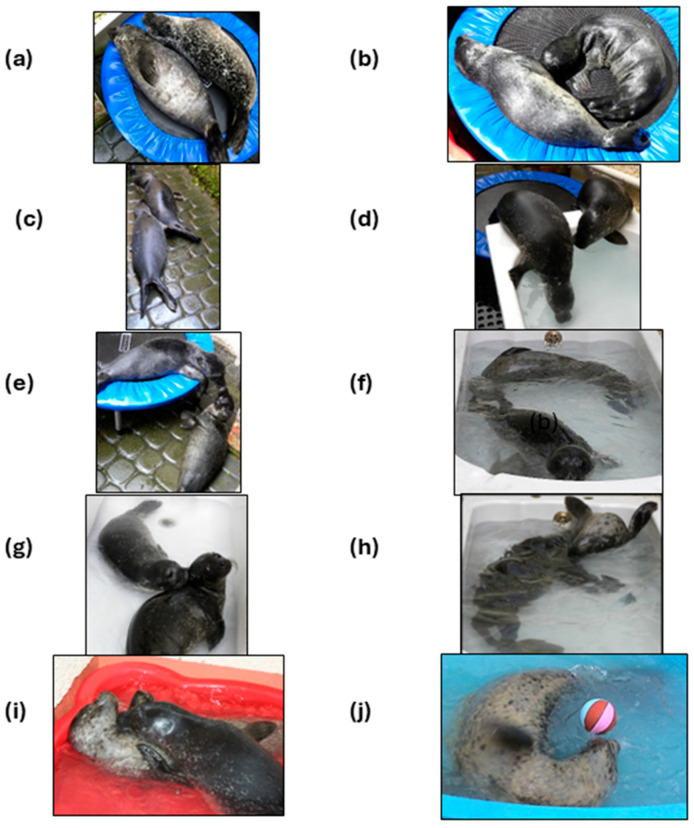
Behaviours recorded. (**a**) Resting on trampoline (C&P d3), (**b**) suckling (U&E d2), (**c**) following (U&E d20), (**d**) orienting to one another while following (U&E d24), (**e**) nose to nose (U&E d3), (**f**) nose-to-hind-flipper contact (C&P d18), (**g**) nose-to-back-of-head/neck contact (U&E d24), (**h**) one pup somersaults over the other while maintaining body contact (U&E d16), (**i**) pup “Piccolo” leans over “Celeste” (2008, d31), (**j**) individual play with a floating ball (“Silver”, 2005 pair, d30) (see also https://www.sealresearch.org/rehab-pups/rehab-diary, accessed on 28 September 2024).

Behaviours that had previously been identified and defined in an ethogram for free-living pups with their mothers (Appendix A; [16]) were used to describe the behaviours of the pup pairs during rehab (Table 2; Figure 3).

Six 24 h samples of CCTV footage at approximately five-day intervals up to rehab day 33 (Table 3) were extracted for the analysis for each rehab pup pair. The data recorded for each 24 h period included the hour before 24:00 and finished at 23:00. The rehab days analysed for C&P were a week later than for the other two pairs (due to the loss of the first week’s recording). All CCTV footage when the carers were in the yard for feeding and cleaning, or when both pups were not visible, was omitted from the data.

The 24 h CCTV footage was sampled by extracting a 15 min period from each hour (Table S1). The pups’ use of each zone was analysed according to whether the pups occupied a zone at any time during that 15 min. As for the previous analysis of free-living pup behaviour [16], the rehab pups’ behaviour was analysed in 15 s segments, according to whether each behaviour occurred (YES or NO) at any time during that 15 s segment. More than one behaviour could therefore occur in any 15 s segment. The exception to this procedure was FOLLOW; this was recorded as a YES if following occurred within 2 min of the first pup moving away. The total number of 15 s segments analysed in each zone with each rehab pair and with free-living mother–pup pairs is given in Table 4.

### 2.4. Quantitative Data Analysis

Following the methodology used to describe the behaviour of free-living pups in the intertidal zone [16], the quantitative data analysis of rehab pup behaviour was based on the proportion of 15 s segments by each pup pair in each zone, in which each behaviour occurred. The k-proportion chi-squared test [52] was used to detect significant quantitative differences in behaviour frequencies in each zone. The chi-squared test was used to compare the proportion of time (% of 15 s segments) the distance between the pups was zero (pups touching), <1 m, 1–2 m, or >2 m.

The data from these three rehab pup pairs were then compared quantitatively with video data previously obtained from the free-living pup study in analogous intertidal zones [16]. For this comparison, the 2-proportion Z-test [52] was used to compare behaviour frequencies by rehab and free-living pups in the *water*, *water’s edge*, and *dry zone* (Table S2).

## 3. Results

### 3.1. Rehab Pup Growth

**Figure 4:**
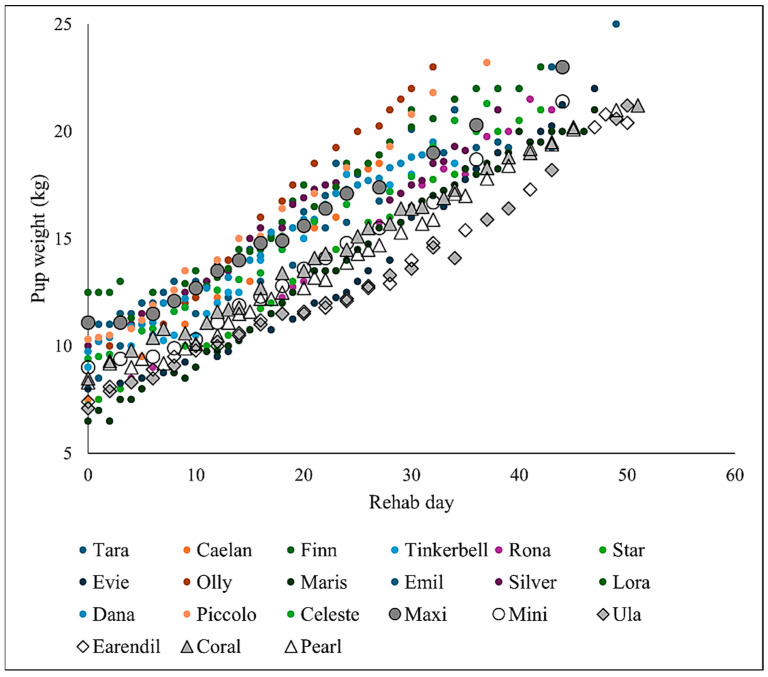
Growth of the six pups of this study (Maxi, Mini, Ula, Earendil, Coral, Pearl) shown with grey, black, and white markers. Growth rates of 15 orphan pups (Tara to Celeste) previously rehabilitated at TSR are shown with smaller coloured dots.

All six pups grew steadily throughout the 7-week rehab period (Figure 4), with an average growth rate of 0.285 kg/day (range: 0.27–0.30 kg/day). Records of growth of 15 previously rehabilitated pups from TSR indicated a similar growth pattern (Figure 4; Table S3).

### 3.2. Time Spent by Rehab Pups in Each Zone

The pup pairs were in the *water* (either pool or bath) for an average of 21% of the total 15 s segments recorded for each pair, 72% in the *dry zone*, and 7% at the *water’s edge*.

**Figure 5:**
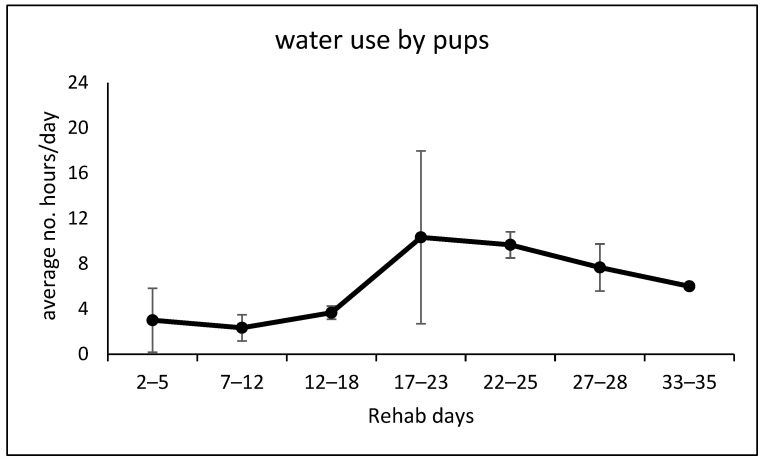
The average number ±SE of hours per 24 hr day during which pups of the three pairs entered the water (see Figure A3).

The pups entered the water less often during the first 16 days in rehab than during days 17–35 (Figure 5; one-tailed *p* = 0.004; *t*-test). The average water hours per day were therefore up to ~15% of time in the first 16 days and up to ~43% thereafter. The pups in their first 2.5 rehab weeks tended to have 2–3 sessions in the water in 24 h, usually not more than 1–2 h per session. From the end of the third week, pups tended to have about three water sessions per day, each of which could last between a few and several hours. Water sessions were generally separated by at least 2 h in the dry zone (Figure A3). The pups all spent some time submerged, either playing together or sleeping below the surface.

The results from the *Flir* thermal imaging for surface body temperature of the pups’ back (Figure A4) when emerging from the water and when fully dry differed by an average of 9.3° (dry: av. 31.4 °C, S.D. = 7.2 °C, *n* = 14; wet: av. 22.2 °C, S.D. = 3.9 °C, *n* = 53). Surface temperatures for other parts of the body are given in Table A1. The wet and dry temperatures for Coral on rehab d0 (23.8 °C and 31.2 °C), when her body mass was 8.6 kg, differed by 7.4 °C.

### 3.3. Distance Between the Paired Pups

The two pups of each pair were almost always close together. All pairs combined were recorded as being <1 m apart in the dry zone for 99.3% of 15 s segments (range: 98.7–100% for the three pairs), 92.6% at the water’s edge (range: 87.7–100%), and 98.1% in the water (range: 97.3–100%). However, the pups spent relatively more time touching (in body contact) in the dry zone (67%) and relatively more time at <1 m (i.e., less than one body length but not touching) at the water’s edge (71%) and in the water (71%) (*p* < 0.0001; chi-squared test).

### 3.4. Description of Behaviours of Pups

The pups usually rested together in the dry zone, on one of the two trampolines provided (Figure 3), although they sometimes rested together in the water after the first 2–3 weeks. When RESTING, their various BODY CONTACT positions included back to back (Figure 3a), venter to back, venter to venter, and head over body. One pup SUCKLING on the other sometimes occurred during rest periods on the trampoline (Figure 3b). The pup being suckled usually tolerated it, infrequently suckled reciprocally, but occasionally flippered at the suckling pup and tried to shift the suckled body area out of reach.

The pups were usually active simultaneously. While active, the pups tended to orient towards one another and FOLLOW their partner when it made DIRECTED MOVEMENT. This orienting was evident in the way in which the pups watched or followed one another as they moved from one end of the yard to the other or climbed in or out of the bath or pool (Figure 3c,d).

NOSING EXCHANGES were an integral part of all the pups’ interactions in all zones, occurring in 24% of all recorded 15 s segments. Nosing contacts occurred both in air (Figure 3e,g) and under the surface (Figure 3f). About a quarter of all nosing contacts were nose to nose (Figure 3e), a quarter were nose to tail/hind-flipper area (Figure 3f), and about half were to the rest of the body, most often the head, neck, and throat area (Figure 3g; Table S1 “body nosing”).

Nose to the lower abdomen area was usually a prelude to suckling and occurred most often in the *dry zone*. Nose-to-nose contacts occurred disproportionately often at the *water’s edge*, while nose-to-hind-flipper contact occurred with disproportionate frequency in the *water*, often as one pup followed the other as it started to haul out. Nose-to-body contacts occurred frequently in all zones, although relatively most often in the *dry zone* (*p* < 0.0001, d.f. = 8; chi-squared test; Table S1 “body nosing”).

**Figure 6:**
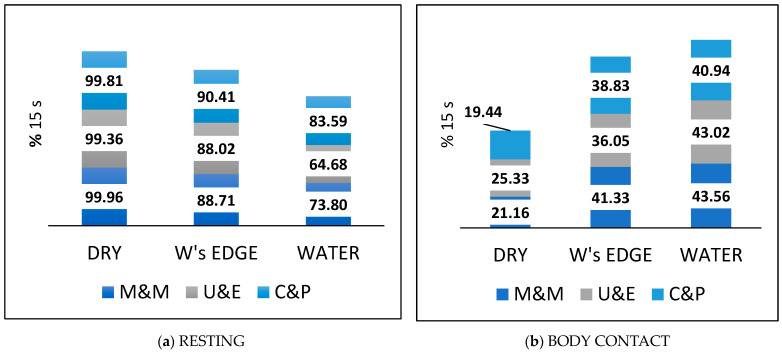

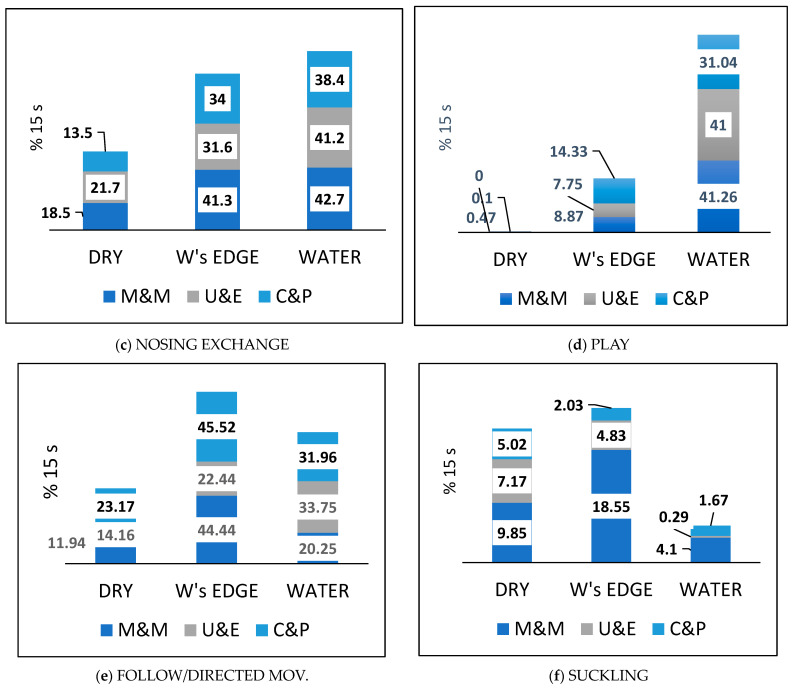
Proportions (as a percentage) of total 15 s segments in each zone in which each behaviour occurred by each of the three pup pairs in rehab.

Over all records of all three pup pairs, behaviour interpreted as PLAY occurred in 9.2% of total 15 s segment records. PLAY occurred most often in the *water* (Figure 6d) and sometimes at the *water’s edge*. The pups typically played interactively as a pair when they entered the water together, with a bout usually lasting about five minutes. In the bath, the interaction consisted of circling after each other, nose to hind flippers (Figure 3f), somersaulting or leaning over each other (Figure 3h,i), or twisting around each other. Contact during play interaction was variously head to head (Figure 3h), head to tail (Figure 3f), or body to body in different configurations, generally involving underwater nose-to-body contact. In the pool, however, physical manoeuvring during pair interaction was limited to two dimensions (Figure 3i). Such paired play accounted for 54%, 49%, and 75% of all play behaviour in the water for M&M, C&P, and U&E, respectively. When the pups had a choice of the bath or pool (U&E and C&P only), they spent 5–6 times more time in paired play in the bath than in the pool (Figure A5). After an energetic paired play bout, the pups would gradually disengage and move more slowly in the water. The play sometimes ended with one of the pups hauling out to rest, while the pup remaining in the water would sometimes attempt to continue the play with its partner by approaching it, often while lying supine or sideways, and making nosing contact. However, if the partner pup failed to respond, the remaining pup sometimes continued with individual play, such as splashing, rapid swimming, or exaggerated movement. Individual play with a ball (occurring relatively infrequently) would involve pushing it around in the water, while rotating the body (Figure 3j).

### 3.5. Frequency of Occurrence of Behaviours in Each Zone

The proportions of total time in each zone spent on each activity were broadly similar between the three pup pairs (Figure 6). When the behaviour frequencies for all three pup pairs were combined, there were significant differences between the zones in the occurrence of each activity. RESTING occurred most often in the *dry zone*, next at the *water’s edge*, and least in the *water* (Figure 6a; *p* < 0.0001; k-proportions chi-squared test). BODY CONTACT and NOSING CONTACTS both occurred the least in the *dry zone*, next at the *water’s edge*, and the most in the *water* (Figure 6b,c; *p* < 0.0001), while PLAY occurred mostly in the *water*, and slightly less often at the *water’s edge* (although the zone difference was not significant), but almost not at all in the *dry zone* (Figure 6d; *p* < 0.0001). Both DIRECTED MOVEMENT and FOLLOWING occurred significantly more often at the *water’s edge* than either in the *water* or in the *dry zone* (*p* < 0.0001), whereas FOLLOWING by one pup as a proportion of DIRECTED MOVEMENTS made by the other pup occurred least often in the *dry zone* (Figure 6e; *p* = 0.004). SUCKLING occurred relatively rarely, and least often in the *water* (Figure 6f; *p* < 0.0001).

### 3.6. Comparison Between Rehab Pup Pairs and Free-Living Pups of Frequency of Occurrence of Behaviours

**Figure 7:**
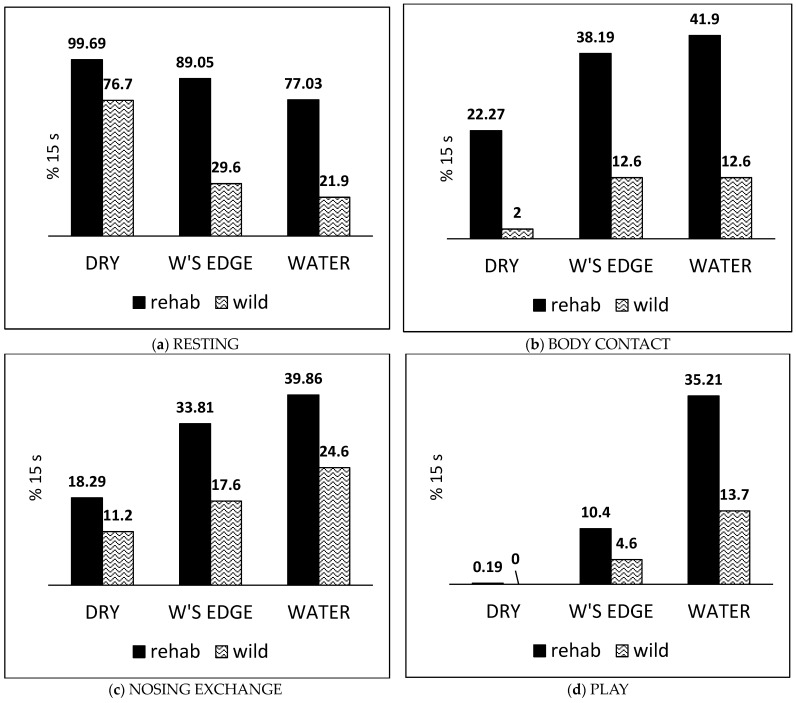

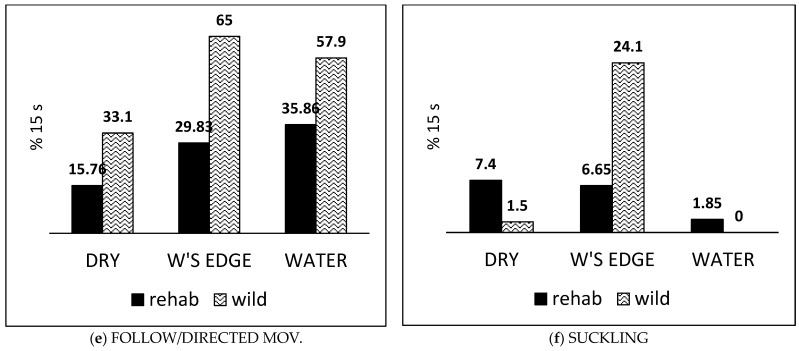
Comparison of proportion (as a percentage) of 15 s segments in which six behaviours occurred between paired pups in rehabilitation (rehab) and free-living pups with their mothers (wild).

**Figure A2:**
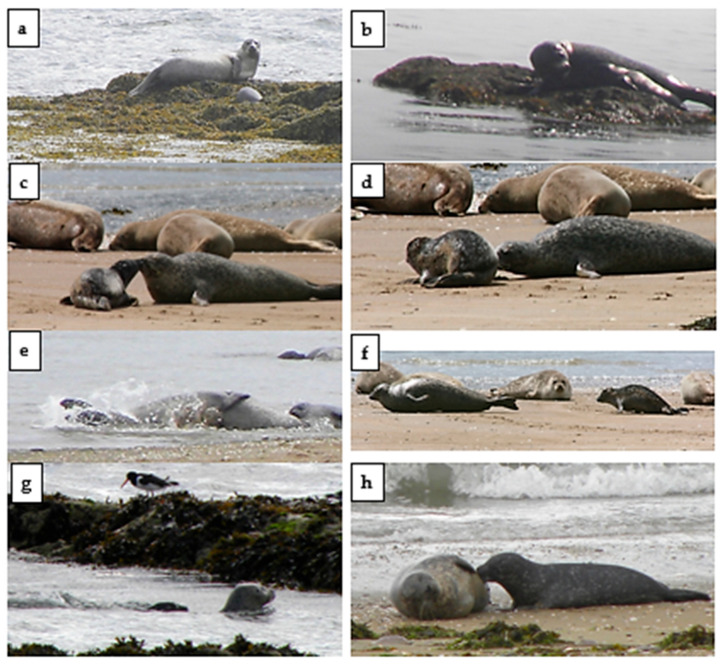
Illustration of behaviours recorded during field study of wild mother–pup pairs [16] (Wilson & Jones, 2018). (**a**) Mother–pup resting, pup asleep ~1 PBL (pup body length) from mother. (**b**) Mother–pup in body contact (mother looking at pup). (**c**) Nose-to-nose contact between mother and pup. (**d**) Mother noses pup’s flank. (**e**) Mother and pup play at water’s edge. Mother is lying on her side, splashing with her hind flippers. Pup is nosing her top/back of head. (**f**) Pup follows mother on beach. (**g**) Pup follows mother in water. (**h**) Pup suckles at water’s edge.

The six behaviours compared between free-living (‘wild’) pups with their mothers and paired pups in rehab were qualitatively similar in all three zones (Figure 7; Figure A2). For both rehab and wild pups, RESTING occurred most often in the *dry zone* and least in the *water* (Figure 7a), while BODY CONTACT and NOSING EXCHANGE occurred least often in the *dry zone* and most often at the *water’s edge* and in the water (Figure 7b,c); most play occurred in the *water* and not at all in the *dry zone* (Figure 7d). However, the quantitative differences between the rehab and wild pups in behaviour frequencies for all behaviours in all zones were significant (*p* < 0.0001, two-proportion Z-test). The rehab pups engaged in relatively more RESTING, BODY CONTACT, NOSING EXCHANGE, and PLAY in all zones than their wild counterparts. The wild pups displayed a higher frequency of FOLLOWING as a proportion of the mother’s DIRECTED MOVEMENT (Figure 7e), and, unlike the rehab pups, they SUCKLED almost exclusively at the *water’s edge* and rarely in the *dry zone* (Figure 7f).

## 4. Discussion

### 4.1. Nutrition and Weight Gain of Rehab Pups

In the past, good welfare of animals in human care has been considered the prevention of negative states, of which those most relevant for orphan seal pups would include hunger, malnutrition, acquired disease, and fear [53,54]. Providing adequate nutrition of neonate harbour seal pups for normal growth of ~0.5 kg/day has been universally challenging for rehab centres in the past. Five of the six pups in this study were probably non-viable in the wild, due to their low birth weight (Table 1) and small body size [1]. However, all six pups, and their 15 predecessors at TSR, all gained 0.27–0.3 kg/day (Figure 4), which is closer to the free-living pup norm (estimated at 0.4–0.6 kg/day [44,55]) than average weight gains recently reported from other centres (0.1, 0.13, 0.21 kg/day [55,56]), and an average of 0.18 and 0.21 kg/day in N. American east and west coast centres, respectively [44]. This relatively rapid weight gain by the TSR pups may have the advantage of reducing chronic levels of the stress hormones prednisolone and prednisone [50] as well as facilitating a relatively early release [42] and was achieved despite the pups being kept out of doors, with no artificial heat, and with free access to water.

### 4.2. Water Use by the Rehab Pups

**Figure A3:**
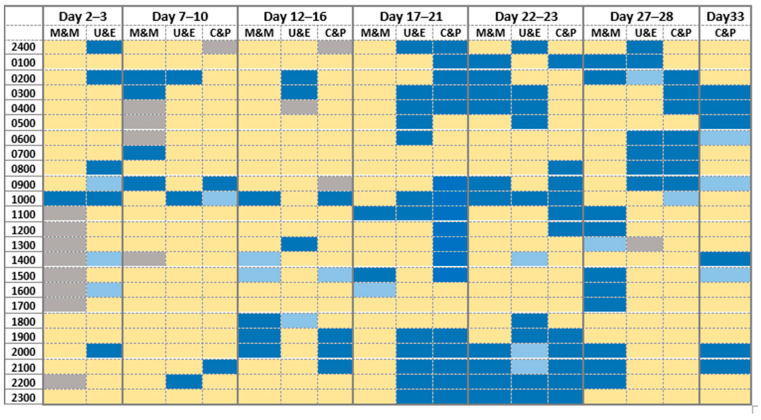
Water use in hour blocks by three orphan pup pairs (M&M, U&E, and C&P) in the present study. Hour blocks indicate if pups entered the water (dark blue), were at the water’s edge (pale blue), or were in the dry zone (yellow). Cells shaded grey indicate no data for those hour blocks.

**Figure A4:**
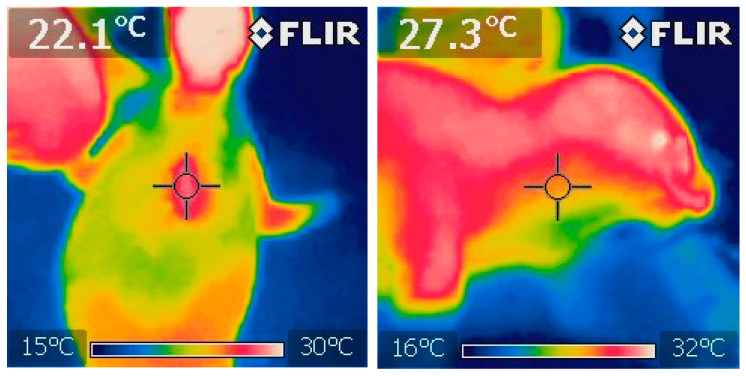
Examples of *Flir* photographs of the back (Left—wet) and side of neck (Right—dry) (Coral, 3 July 2016).

The factors affecting the orphan pups’ decision to enter or leave the water are not known, but may include individual variation, weather, or feeding and yard cleaning schedules as well as the pups’ physical growth; in our data, the wide SE for days 17–21 (Figure 5) is due to pups M&M apparently not increasing their time in the water—at least on sample days—until days 22–23 (Figure A3).

Free-living pups with their mothers show an increase in time in the water, from ~35% up to ~80% [10,11]. This is more than the ~15% up to ~43% recorded for our rehab pups. However, for free-living pups, there would be a greater need to be in the water for longer as their haul-out site may become covered by an incoming tide or they may accompany their mothers on a foraging trip offshore [7,15]. The latter three CCTV samples from our rehab pup recordings (days 22–35) approximate the post-weaning period in the wild, when pups spend almost all their time in the water while they are beginning to explore independently and learning to forage [20].

Our thermal imaging of C&P, comparing their body surface temperatures when dry versus emerging from the water, indicated that they had adequate thermal homeostatic regulation, which is thought not to be a source of major energy expenditure, suggesting that there is no need to restrict young pups to a daily limited swim [51], since the present study indicates that even underweight neonate pups, in rehab circumstances where they can enter and leave the water unaided, are capable of self-regulating their time in the water and thus avoiding hypothermia. Their frequent submergence, while playing or sleeping, should help to build the pups’ blood oxygen storing capacity, facilitating their aerobic diving capacity after release [45].

Thus, although stress hormone studies of pups in rehab [2,50,57] may reflect negative states or the absence thereof, assessment of positive emotional states requires behavioural assessment. It is generally understood that positive welfare requires “an appropriate environment for resting and activity, and sufficient space, proper facilities, and the company of the animal’s own kind” [53]. For orphan harbour seal pups in rehab, this means a captive environment that can mimic the essential features of the free-living harbour seal pup’s social environment and intertidal habitat, as in the present study, i.e., a conspecific (substituting for the mother), an area of shallow water, and a dry haul-out area.

The pups’ free water access indeed enabled them to display all the behaviours, including social contact and play, previously described as filial behaviours for free-living pups with their mothers and peer behaviours between pups [16,20]. A previous study indicated relatively low chronic levels of the “stress” hormone cortisol in pups with free water access compared with pups kept without water [50] and suggested that behavioural observations would be needed for a better understanding of indicators of positive welfare. The behaviour observations of the present study have been able to demonstrate enhanced levels of behaviour indicators of positive affect (PLAY, BODY CONTACT, FOLLOWING, and NOSING EXCHANGE) when the pups were in the *water* compared with levels of these behaviours in the *dry zone* (Figure 6b–e).

**Figure A5:**
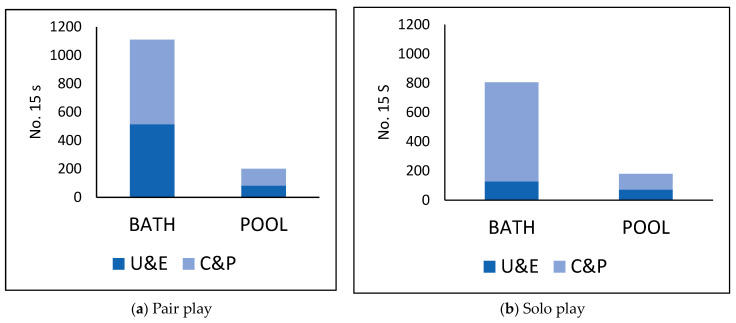
Pups’ preference for bath over pool for play: the total no. 15 s segments in bath or pool for each pup pair.

The pups’ activity periods typically started in the water, with vigorous social play, thought to be an indicator of good health and welfare [58] as well as having a beneficial effect on infant brain development [59]. Although the pups M&M did not have the option of the deeper water (~35 cm) of the bath, they nevertheless spent the same proportion of time in aquatic play as the other two pups. However, the pups U&E and C&P had a choice of the pool and bath for play, and clearly chose the bath (Figure A5), which had sufficient water depth for three-dimensional physical manoeuvring. The pups’ choice of the bath over the pool for play is instructive for the future design of pens for pups in the early weeks of rehab.

### 4.3. Behaviours of Rehab Pup Pairs Compared with Pups in the Wild

Because the behaviours of the pup pairs resembled the behaviours of mother–pup pairs in the wild, we consider that the paired orphan pups in rehab were acting to some extent as mother substitutes for one another. The observed quantified social interactions considered to be indicative of mutual positive affect for both pups were BODY CONTACT, NOSING EXCHANGE, and PLAY. FOLLOWING and SUCKLING were thought to be positive for at least the following or suckling partner. We had previously observed qualitatively similar positive behaviours by six previous orphan pup pairs in rehab (https://www.sealresearch.org/rehab-pups/rehab-diary; accessed on 22 September 2024) and between Maxi and Mini on the shore, as well as reports of apparent bonding between other stranded pups before rescue [1]. We therefore consider that the potential for development of this pair relationship between two pups may be typical for harbour seal orphans endeavouring to compensate for their mothers’ absence.

A striking quantitative difference between the rehab and the wild pups is that the rehab pups spent more time in body contact in all zones than the wild mother–pup pairs, with the difference in the *dry zone* being particularly striking (Figure 7b). When in contact, the pair’s mutual sensory stimulation via the thermal and afferent cutaneous tactile nerves from the skin likely involves neurotransmitter systems including opioids, dopamine, and noradrenaline [60] as well as oxytocin release, signalling a positive message of social attachment and security [61]. The disproportionately high amount of nosing contact while resting in the *dry zone* suggests that this may have enhanced the pups’ sense of security. 

The gentle touch of hairy skin is thought to stimulate *C-low threshold mechanoreceptors* (CLTMs), which become more sensitive when the hair and skin are wet [23,62,63]. This phenomenon may be part of the reason why contact play for both our rehab pups and wild pups occurs only when the pups are in the water or wet at the water’s edge [16], and wild adult harbour seals are very much more tactile when they are wet than when they are dry [23].

### 4.4. Rehab Pup Contact Behaviours and Social Bonding

Harbour seal pups in rehab are usually fed liquid formula via stomach gavage because they will rarely suckle from a baby’s bottle. However, another pup’s warm skin, coat, and odour appear to provide sufficient stimuli for suckling, and therefore it may compensate psychologically to some small extent for the lack of normal nutritive suckling from the mother. “Cross-suckling” may be considered a problem with paired dairy calves [24], or harbour seal pups after a longer period in rehab [64]. However, the cross-suckling by our rehab pups was relatively infrequent (Figure 6f), and—even though the suckled pup did not always appreciate the contact—we consider it to be positive for the welfare of these young orphan pups rather than a problem. 

The overall occurrence of play recorded in 9.2% of observation time of the rehab pups and 4.4% for the free-living pups with their mothers falls within 1–10% of the total time budget noted in nearly every species studied [65]. The aquatic paired play enhances olfactory and tactile contact and resembles mother–pup and juvenile play [66,67] as well as adult dyadic play and courtship in harbour seals [68,69]. This manner of physically gentle social play seen in many mammal species is thought to foster a positive and harmonious social relationship between the participants [65]. The greater body contact and nosing contact levels seen between the rehab pups—even when dry—compared to the wild pups may enhance bonding in compensation for the mother’s absence. Nevertheless, the relatively greater amount of play by the paired rehab pups compared with the free-living pups with their mothers may, by analogy to domestic cat studies, be in part a response to the absence of maternal proximity and care [70].

**Figure A6:**
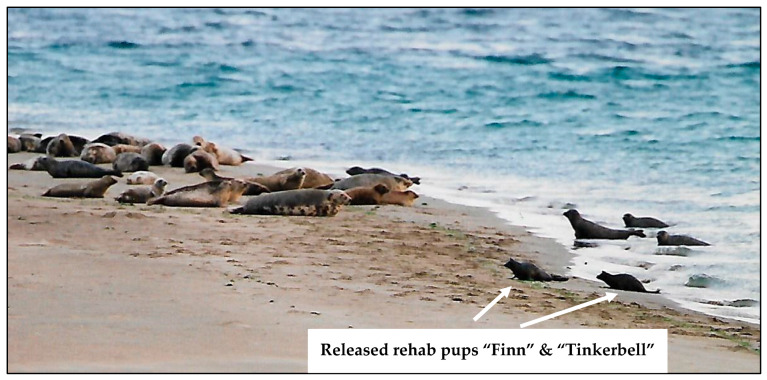
Two paired pups (“Finn” and “Tinkerbell”, wearing head-mounted VHF tags) after release near the haul-out site (Dundrum Bay, Co. Down, north-east Ireland). The pup pair swam together from their release site down the estuary channel and hauled out together beside the seal colony. It is thought that integrating with the local seal colony after release will facilitate the rehab pups’ development of normal post-weaning behaviour of free-living pups of a similar age (~7 weeks).

The harbour seal pup’s following response to its mother has been considered an integral part of mother–pup bonding and likened to “imprinting” [71]. Our rehab pups displayed a following response to one another, albeit less strongly than the wild pups with their mothers, additionally suggesting that conspecific “imprinting” and social bonding occur between rehab pups housed socially. It may be argued that while the pups of a pair evidently mutually enjoy the companionship, determining whether the pups need the partner pup in the way that a free-living nursing pup needs its mother requires a “demand” test, where a pup separated from its partner is required to exert measurable effort to reunite [72,73,74]. Preliminary tests involving separating two cohabiting pups by a barrier found that the separated pups immediately started to make strenuous efforts to regain contact (unpublished data; https://www.sealresearch.org/files/harbour%20seal%20orphan%20pup%20in%20rehab%20with%20companion%20pup%20.pdf, accessed on 21 September 2024). Their efforts to reunite appeared equivalent to temporarily separated free-living pups trying to find or follow their mothers and imply that paired orphan pups do indeed act as mutual mother substitutes. Our paired pups’ social bonding may help them integrate with a colony—as exemplified by paired pups “Finn and Tinkerbell” who, when released, followed each other directly into a haul-out group (Figure A6; https://www.sealresearch.org/rehab-pups/rehab-diary/2000/release, accessed on 21 September 2024).

### 4.5. Prospects for Rehab Orphan Pups After Release

The behavioural demands on a newly weaned pup or newly released rehabilitated pup may be complex and varied and require behavioural flexibility—not only must they learn the identities of other seals making up their local social group or local population, but they must also develop understanding of the topography and geography of their haul-out sites [38], combined with understanding the diurnal and seasonal movements of fish [35,36], and the effects of tidal ebb and flow on foraging and resting habitats. Free-living pups with their mothers and proximity to other seals will not only have species-normal neural development but will also have a head start on acquiring all this information via their early experience of both the physical and social environment. Observations of pups with their mothers and at weaning suggest how their social following response around and to and from their haul-out site may provide social facilitation to enhance the exploration and development of successful foraging tactics [16,20]. Although rehabilitators cannot provide any of this experience for orphan pups during captive care, they may be able to provide substitute pup–pup socialisation, which may help to normalise post-natal brain development and thus ease their transition after release to the complex marine environment.

## 5. Conclusions

This study has been able to show how housing orphan harbour seal pups in pairs with water access gives them the opportunity to engage in social contact and play behaviours, which are generally accepted as indicators of positive affect. We are therefore proposing that cohabitation for neonate harbour seal orphans during the early stages of captive care should minimise the risk of later socialisation deficits and cognitive impairment while promoting their positive emotion during rehab.

The observations and proposals here apply specifically to orphan pups admitted as neonates or very young nursing pups. Harbour seal pups stranding at a post-weaning stage will already have had normal post-natal socialisation with their mother and some experience of the marine environment and will therefore have different needs in captive care from a stranded neonate. Pups admitted as neonates, but spending longer time in captive care than the pups in this study, will also bring different challenges, such as the development of stereotypical behaviour. Also, the young of other pinniped species will have varying needs according to their species developmental timetable. Nevertheless, the principle should still apply of tailoring the design of captive care to the pup’s stage of development and its previous experience in relation to the behaviour of its free-living peers.

Our proposals apply only to pups that appear overtly healthy apart from their “orphan” status and likely low body weight; orphan pups suffering from pneumonia, elevated temperature, or known infectious diseases should be maintained separately until they have recovered. However, because of the welfare benefits to harbour seal pups of cohabiting with water access, evident from the present study, we suggest that policies of isolating pups in quarantine should be interpreted flexibly, according to circumstances.

To summarise, the design of seal pup rehabilitation systems should ideally be based on integrated information from observations of seal pups in the wild, research on the implications of maternal separation in domestic and laboratory species, and results from behaviour experiments with seals in captivity. The challenge for seal pup care centres is therefore how to adopt a precautionary approach with the aim of minimising the likely negative effects of social isolation while still complying with veterinary precautions on transmittable diseases and national policies on seal rehabilitation.

## Figures and Tables

**Table 1:** Pup stranding and rescue.

Pup	Sex	Body Mass at Stranding (kg)	Date (dd/mm/yy)	Context of Rescue
Maxi	M	11.1	19/07/13	These two pups were reported by local residents as having been moving in and out of the water together, but away from the haul-out group, for 1–2 days before stranding on beach >100 m from haul-out group.
Mini	F	9.0		
Ula	F	7.1	05/07/14	Found by local resident on mainland shore, far from haul-out group on island; pneumonia diagnosed, treated with antibiotic.
Earendil	M	7.4	07/07/14	Found by local resident in grass beside outbuilding approx. 100 m above high-tide mark.
Coral	F	8.5	03/07/16	Second day observed without mother; left alone on ebb tide, suckling on hind flippers, distress calling.
Pearl	F	8.3	05/07/16	Alone at ebb tide, asleep in hunched posture, unresponsive to approach and when placed in net. Effectively blind in one eye and believed to have some neurological impairment. Vomiting episodes on days 4 and 19, treated with antibiotic and omeprazole.

**Table 2:** Behaviours of the rehab pup pairs described and quantified (c.f. behaviours of free-living mother–pup pairs recorded and analysed; [16]).

Observed Behaviour	Description
*RESTING*	One or both pups lie on shore or at surface of water, no voluntary forward movement, may be asleep or alert
*BODY CONTACT*	Any part of the body in contact between the paired pups or another pup in addition to nosing contact
*NOSING EXCHANGE*	Muzzle-to-muzzle contact between the two pups; one pup making nose or muzzle contact with any part of the partner pup’s body
*PLAY*	Vigorous or exaggerated body or locomotor movement, with relaxed body tone. Includes social play by the pup pair and individual play with an object
*FOLLOW/DIRECTED MOVEMENT*	One pup follows partner pup when it moves forward
*SUCKLING*	One pup suckles on the other

**Table 3:** Dates of CCTV recording analysed for the present study for each pup pair (for pairs U&E and C&P, the 2nd pup arrived two days later than the 1st pup).

M&M (Rehab Day)	U&E (Rehab Day of 2nd Pup)	C&P (Rehab Day of 2nd Pup)
21/07/2013 (d2)	10/07/2014 (d3)	15/07/2016 (d10)
26/07/2013 (d7)	15/07/2014 (d8)	21/07/2016 (d16)
31/07/2013 (d12)	20/07/2014 (d13)	26/07/2016 (d21)
05/08/2013 (d17)	25/07/2014 (d18)	28/07/2016 (d23)
10/08/2013 (d22)	30/07/2014 (d23)	02/08/2016 (d28)
15/08/2013 (d27)	04/08/2014 (d28)	07/08/2016 (d33)

**Table 4:** Total no. of 15 s segments analysed from CCTV recording of rehab pup pairs (this study) and video clips of free-living pups with their mothers (from Wilson and Jones, 2018 [16]).

	**ZONES**		
	DRY	W’s EDGE	WATER
**Rehab pairs**			
M&M	8075	496	1561
U&E	9675	993	1727
C&P	7334	886	4153
Total, all pairs	25,084	2375	7441
**Free-living pups**			
Pups with mothers	1422	1966	671

## Data Availability

Data supporting the reported results may be found in the Supplementary Materials.

## Supplementary Materials

The following supporting information can be downloaded at https://www.mdpi.com/article/10.3390/ani14223264/s1, Table S1: M&M-U&E-C&P-datasets.xlsx; Table S2: Behaviours rehab & rehab vs. wild—15-s summaries.xlsx; Table S3: Rehab pup growth rates all Tara to Pearl.xlsb.

## Appendix A

The present study of the behaviour of harbour seal orphan pups in captive care for rehabilitation was based on the methods and results of a field study by Tara Seal Research (TSR) of the breeding colony in Dundrum Bay, north-east Ireland (reference [16] in main text). Five of the six orphan pups in the present rehab study at TSR were stranded in Dundrum Bay; the sixth (Ula) was stranded at Strangford Lough (Figure 1).

The field study covered six pupping seasons (2010–2015) at two pupping sites in Dundrum Bay, north-east Ireland. Behaviour was video-recorded from vantage points out of sight of the seals. Mother–pup pairs at the site were recorded in turn, each for 5 min unless they disappeared underwater before then. Each video clip was divided into sequential 15 s segments. Each 15 s segment was classified according to one of three habitat zones: the *water*, *water’s edge*, and *dry zone*. Behaviours were recorded in binary form according to whether they did or did not occur in a 15 s segment. The pup behaviours analysed were RESTING, SUCKLING, PUP FOLLOWS/MOTHER’S DIRECTED MOVEMENT, and PLAY (solo or with mother). The behaviours of mothers and pups were NOSING EXCHANGE (nosing contacts by either mother or pup) and BODY CONTACT. The DISTANCE between a pup and its mother was estimated in pup or adult body lengths (PBLs or ABLs). These behaviours were illustrated photographically by Wilson and Jones [16] and illustrated again here (Figure A1) using another set of photographs. In the present study of orphan pup pairs in rehab, the behaviours of the two pups, analogous to each of the pup–mother behaviours, were recorded by continuous CCTV with samples of the recording analysed instead of short video clips obtained with hand-held video cameras.

For the wild pup–mother pairs, behaviours recorded were analysed according to the proportion of the total 15 s segments in which each behaviour occurred in each habitat category (k-proportions chi-squared test). For the present study of orphan rehab pups, the same k-proportions test was used to compare behaviours in each of the three habitat zones and the two-proportions Z-test was used to compare the behaviours of the rehab pup pairs with the wild pup–mother pairs in each habitat zone.

### Appendix A.1. Thermal Imaging of Coral and Pearl When Wet and Dry

**Table A1:** Average surface body temperatures when wet and dry (Coral and Pearl).

	Wet			Dry		
	*n*	Av.	S.D.	*n*	Av.	S.D.
Nostrils	41	20.63	3.03	11	29.9	3.70
Side of neck	27	22.30	2.32	14	28.75	4.20
Flank	17	21.19	3.02	11	30.26	7.20
Back	53	22.18	3.90	14	31.43	7.17
Hind flipper	45	19.23	5.18	17	33.52	3.83
Fore flipper	27	21.12	5.87	10	33.47	2.30
